# A study on the structural relationship among dance training experience, motor imagery, cognitive flexibility, and emotion regulation strategies in young adults

**DOI:** 10.3389/fpsyg.2026.1956871

**Published:** 2026-09-04

**Authors:** Lei Li

**Affiliations:** School of Pre-school and Art Education, Zhoukou Vocational and Technical College, Zhoukou, China

**Keywords:** cognitive flexibility, cognitive reappraisal, dance training experience, embodied cognition, mediation analysis, motor imagery, young adults

## Abstract

**Introduction:**

Based on embodied cognition theory, this study investigates the structural relationship among dance training experience, motor imagery, cognitive flexibility, and core emotion regulation strategies in young adults. It also examines the statistical mediating role of cognitive flexibility in the association between motor imagery and cognitive reappraisal.

**Methods:**

A cross-sectional survey design was adopted to assess 1,070 young adults aged 18 to 35. Core variables were measured using standardized scales for motor imagery, cognitive flexibility, and cognitive reappraisal. Data analysis included descriptive statistics, Welch's independent samples t-test, Pearson correlation analysis, hierarchical multiple regression, and statistical mediation analysis based on 5,000 Bootstrap resamples.

**Results:**

Participants with regular dance training experience scored significantly higher in motor imagery, cognitive flexibility, and cognitive reappraisal than those without dance training experience, with respective differences of *d* = 0.594, 0.350, and 0.234 (all *p* < 0.001). Motor imagery, cognitive flexibility, and cognitive reappraisal were significantly and positively correlated with each other, with correlation coefficients ranging from 0.369 to 0.458 (all *p* < 0.001). Within-group analysis revealed that motor imagery ability showed a significant gradient increase with training intensity and duration, whereas higher-order cognitive and emotional differences primarily existed as a binary distinction between the experienced and inexperienced groups. Mediation analysis showed that cognitive flexibility provided a significant indirect statistical linkage between motor imagery and reappraisal, accounting for 34.11% of the total statistical relationship (95% CI = [0.091, 0.182]).

**Discussion:**

In young adults, dance training experience is associated with higher motor imagery, cognitive flexibility, and cognitive reappraisal abilities. This study supports a theoretical linkage from bottom-up sensorimotor representation to top-down emotional reconstruction, providing an innovative perspective for integrating embodied movement practices to foster physical-psychological synergy and promote youth emotional wellbeing.

## 1 Introduction

Young adulthood is an important developmental stage in which individuals gradually progress toward independence in psychological, social, and occupational functioning. During this period, individuals often need to cope with multiple pressures such as academia, employment, interpersonal relationships, and social role transitions. The ability to effectively regulate emotional experiences triggered by stressful events is a core factor affecting the psychological adaptation and long-term health of young adults. Among various emotion regulation strategies, cognitive reappraisal is representative as an effective antecedent-focused regulation strategy. It mainly refers to individuals adjusting the emotional meaning of an event by changing their understanding and interpretation of the emotional event, thereby improving subsequent emotional experiences (Gross, 1998; Gross and John, 2003). Considering that cognitive reconstruction is the core foundation of multiple adaptive emotion regulation strategies, this study focuses on selecting cognitive reappraisal as the observation indicator for emotion regulation strategies.

Traditional emotion regulation research mainly explains the individual differences in cognitive reappraisal from information processing processes such as attention, memory, appraisal, and cognitive control. In recent years, embodied cognition theory has further proposed that higher-order cognitive and emotional processing are not completely independent of the body, but are closely related to the individual's sensorimotor experiences and their continuous interaction with the environment (Barsalou, 2008; Wilson, 2002). From this perspective, physical activity involves not only explicit movements but may also be connected to higher-order psychological processes such as action representation, attention shifting, and situational interpretation. Furthermore, recent multi-level theoretical frameworks point out that comprehensive leisure activities, such as dance, not only involve physiological arousal but also systematically promote individual mental wellbeing through interactive psychological (e.g., emotion regulation), social, and behavioral mechanisms (Fancourt et al., 2021).

Dance is a comprehensive physical activity that typically involves action sequence memory, spatial orientation, rhythmic synchronization, postural control, action monitoring, and sensorimotor feedback simultaneously (Karpati et al., 2015). In dance learning, participants need to continuously form action representations, predict outcomes, and adjust actions based on feedback. Existing studies have found that individuals with experience in complex movement training often show specificities in action observation and motor imagery (Bläsing et al., 2012; Calvo-Merino et al., 2005), suggesting that dance training experience may be closely related to an individual's motor imagery ability (Mao et al., 2022).

Motor imagery refers to the process in which an individual mentally simulates an action and its sensory consequences without actually executing the action. Motor imagery can involve different forms, such as external visual imagery, internal visual imagery, and kinesthetic imagery, and its vividness reflects the individual's subjective ability to maintain and manipulate action representations at the psychological level (Roberts et al., 2008). Clearer motor imagery involves the maintenance and reorganization of action information, and these processing procedures are theoretically considered to be connected with generalized cognitive control and cognitive flexibility.

Cognitive flexibility refers to an individual's tendency to adjust cognitive modes according to situational changes, generate alternative explanations, and shift between different options (Dennis and Vander Wal, 2010). Some neural reuse literature suggests that the deployment of motor imagery and cognitive control may share executive resources in the prefrontal and parietal lobes (Anderson, 2010). At the same time, cognitive flexibility also provides preconditions for the smooth implementation of cognitive reappraisal: individuals with higher cognitive flexibility are more likely to break free from a single interpretation of negative events and generate alternative understandings, thereby implementing cognitive reconstruction more smoothly.

Although previous literature has separately explored local relationship segments such as movement training and imagery, cognitive shifting and emotional reappraisal, few studies have systematically examined the multi-level structure of young adults' dance training experience, motor imagery, cognitive flexibility, and emotion regulation strategies within the same large-sample network. Unlike previous single-path studies that only explored local relationship segments, this study for the first time constructs a complete structural model encompassing “sensorimotor layer, executive function layer, and emotion regulation layer” in a large sample group. Accordingly, by comparing the inter-group differences among those with and without regular dance training experience, and using multiple regression and mediation models to analyze the statistical association pathways between variables layer by layer, this study aims to reveal the structural associations from basic sensorimotor representations to higher-order cognitive adaptability, and then to emotion regulation mechanisms. This research helps deepen the understanding of the linkage rules between sensorimotor representation and higher-order cognitive emotions from the perspective of embodied cognition.

## 2 Literature review and research hypotheses

### 2.1 Dance training experience and core psychological variables

Dance practice requires participants to form precise action representations after observing demonstrations and continuously revise action schemas in practice. Motor imagery serves as a bridge in this transition. A large number of empirical surveys of professional athletes and performers indicate that groups with complex motor control experience can often accomplish internal action simulations more efficiently. Furthermore, long-term participation in dance activities requires integrating environmental feedback, such as spatial positioning and collaboration with others, to continuously adjust expectations, which echoes the cognitive processing mechanism of individuals suppressing dominant responses and being flexibly adaptable. In the emotional dimension, the musical rhythm and physical release of dance activities may also be positively associated with the daily usage preferences of individuals' emotion regulation strategies (Basso et al., 2021). Based on this, this study proposes:

**H1:** Young adults with regular dance training experience have significantly higher average scores in motor imagery, cognitive flexibility, and cognitive reappraisal than those without dance training experience.

### 2.2 The relationship among motor imagery, cognitive flexibility, and cognitive reappraisal and its mediation mechanism

Motor imagery is not only the representation of static visual images but involves the dynamic simulation of sequences, spatial positions, and muscle exertion. Mentally completing the spatial rotation of movements and the free switching of perspectives inherently requires abundant working memory and the ability to suppress mental sets, which theoretically overlaps highly with the intrinsic requirements of cognitive flexibility (Diamond and Ling, 2020). Recent neuroscientific reviews indicate that cognitive and behavioral flexibility highly depend on the dynamic interaction of the frontoparietal and salience networks, and the internal simulation of complex actions serves as a typical mechanism to activate and reuse these higher-order executive networks (Uddin, 2021). Since both maintaining dynamic motor representations and switching cognitive strategies demand shared executive control resources, a higher level of motor imagery ability is often closely related to an individual's flexible shifting among multiple cognitive representations and task requirements.

Building on this, the capacity for flexible shifting in the cognitive dimension provides key preconditions for higher-order emotion regulation. The core challenge of implementing cognitive reappraisal lies in the fact that when an emotional stress event occurs, individuals are easily confined to a single negative interpretation framework due to stress reactions. An important prerequisite for overcoming this limitation is that the individual has a high level of psychological capacity to resist cognitive rigidity. Facing setbacks, individuals with high levels of cognitive flexibility can more effectively construct alternative interpretive frameworks (for example, reframing a loss as an opportunity for reflection), thereby smoothly completing cognitive reconstruction.

Based on the above theoretical framework, motor imagery may not only be directly associated with emotion regulation ability, but may also indirectly support the implementation of cognitive reappraisal strategies by acting through the bridging role of cognitive flexibility. Accordingly, this study proposes the following hypotheses:

**H2:** Motor imagery, cognitive flexibility, and cognitive reappraisal are significantly and positively correlated with each other.
**H3:** Cognitive flexibility demonstrates a significant statistical indirect effect in the relationship between motor imagery and cognitive reappraisal; after incorporating cognitive flexibility, the direct effect of motor imagery on cognitive reappraisal remains significant.

## 3 Research methods

### 3.1 Subjects and procedures

This study adopted a cross-sectional questionnaire design, with data collection conducted between February and April 2026. Recruitment was carried out through an online platform using a combination of convenience and snowball sampling. Inclusion criteria were: aged between 18 and 35, able to independently understand the questionnaire, and sign an informed consent form. Exclusion criteria included: missing data due to early withdrawal; total response time below the normal reading threshold; and presenting a straight-line or highly regular abnormal response pattern (such as blindly selecting the same option for reverse-scored items).

Using G^*^Power 3.1 software (Faul et al., 2007) for estimation (setting a medium effect size, α = 0.05, statistical power 1- β = 0.95), the minimum sample requirement was 400. To control the impact of data attrition, a total of 1,120 questionnaires were collected in this survey. After strict cleaning, 50 invalid questionnaires were excluded, finally yielding 1,070 valid and complete samples (effective rate 95.54%). The average age of the participants was 26.18 years (*SD* = 5.30). This sample size, which far exceeded the minimum requirement, provided sufficient statistical power for subsequent complex multivariate statistical models and fine-grained subgroup analyses.

### 3.2 Measurement tools

The complete questionnaire and underlying data coding rules are detailed in Supplementary materials 1, 2.

Demographics and Dance Training Experience: Variables included gender, age, and highest educational attainment. Educational attainment was divided into 4 levels from “high school and below” to “master's degree and above”. Dance training experience was self-assessed based on individuals' daily activity habits. Rather than imposing a strict numerical threshold for weekly frequency or historical duration, “regular dance training” was qualitatively operationalized as self-reported engagement in structured practices (e.g., contemporary, ballet, or hip-hop) within formal or semi-formal settings. Accordingly, participants were classified into two groups: those with and without such training experience. For the subject group reporting experience, their training frequency and duration were further collected. The cumulative training duration was divided into four levels from “less than 1 year” to “more than 5 years”, and the training frequency in the past six months was divided into four levels from “occasionally (less than once a week)” to “five times or more per week”.

Motor Imagery: The Vividness of Movement Imagery Questionnaire-2 (VMIQ-2) developed by Roberts et al. (2008) was adopted. To minimize participant fatigue in a large-scale survey while preserving robust construct validity, we adopted a validated brief 6-item version covering two dimensions: external visual imagery (e.g., “imagining myself jumping forward continuously”) and kinesthetic imagery (e.g., “imagining the feeling of calf and foot muscle contraction and force generation at the moment of jumping upward”). The items were translated into Chinese using a standardized back-translation procedure, and the semantic equivalence of the Chinese and English versions was confirmed during the pre-survey stage. Responses were measured on a five-point Likert scale (one = *no image/sensation at all*, five = *perfectly clear and vivid*). All items were forward-scored, with higher scores indicating clearer imagery ability. The Cronbach's α coefficient for this module in this sample was 0.814.

Cognitive Flexibility: The Cognitive Flexibility Inventory (CFI) developed by (Dennis and Vander Wal 2010) was selected. Similarly, to optimize completion rates while successfully retaining the core construct validity of the original instrument, a validated simplified seven-item version was utilized. This version mainly covers the ability to seek alternative solutions (four forward items, e.g., “I usually consider multiple possible options before making a decision”) and features controlling cognitive rigidity (three reverse items, e.g., “When encountering setbacks, I often get stuck in a ‘dead end”'). These items also underwent independent back-translation evaluation and Chinese context adaptation proofreading. Including reverse-scored items (using a 6–X conversion method), scoring ranged from one (*strongly disagree*) to five (*strongly agree*). Higher scores reflect better cognitive shifting ability and mental flexibility. The Cronbach's α coefficient for this module in this sample was 0.773.

Emotion Regulation Cognitive Reappraisal: The Emotion Regulation Questionnaire (ERQ) developed by Gross and John (2003) was selected. This study directly adopted the Chinese revised version heavily tested for reliability and validity and translated by Wang et al. (2007), extracting its cognitive reappraisal subscale (containing six items). This subscale mainly assesses individuals' behavioral tendencies to change their perspective to alter emotional experiences (e.g., “When I want to feel less negative emotion, I change the way I'm thinking about the situation”). A seven-point Likert scale from one (strongly disagree) to seven (strongly agree) was used, with higher scores representing more frequent use of cognitive reappraisal strategies in daily life. The Cronbach's α coefficient for this module in this sample was 0.852.

### 3.3 Data analysis strategy

Data preprocessing, descriptive statistics, internal consistency testing, mean testing, and hierarchical multiple regression analysis were completed based on the IBM SPSS Statistics 26.0 software module; mediation effect analysis was implemented using the PROCESS v4.0 (Model 4) macro program. The steps included: (1) Treating age as a continuous numerical value, encoding gender as zero = *male*, one = *female*, and measuring education on an ordinal scale according to one = *high school and below* to four = *master's degree and above*; (2) Screening for potential outliers based on standardized *Z*-scores and evaluating the potential risk of common method bias through Harman's single factor analysis; (3) Addressing potential variance heterogeneity among groups, Welch's independent samples *t*-test was used to compare differences in core variables across different dance training experience groups; (4) Calculating Pearson correlation coefficients between variables and constructing hierarchical multiple regression models to explore the statistical predictive value of independent variables on the dependent variable after controlling for demographic variables such as gender, age, and highest education; (5) Applying the Bootstrap method to calculate the indirect effect estimate of cognitive flexibility (setting 5,000 resamples and reporting the standardized mediation coefficient along with the 95% corrected confidence interval; significance is determined if it does not contain 0). The significance level for all tests was set at α = 0.05.

## 4 Data analysis and results

### 4.1 Data preprocessing and common method bias test

**Figure 1:**
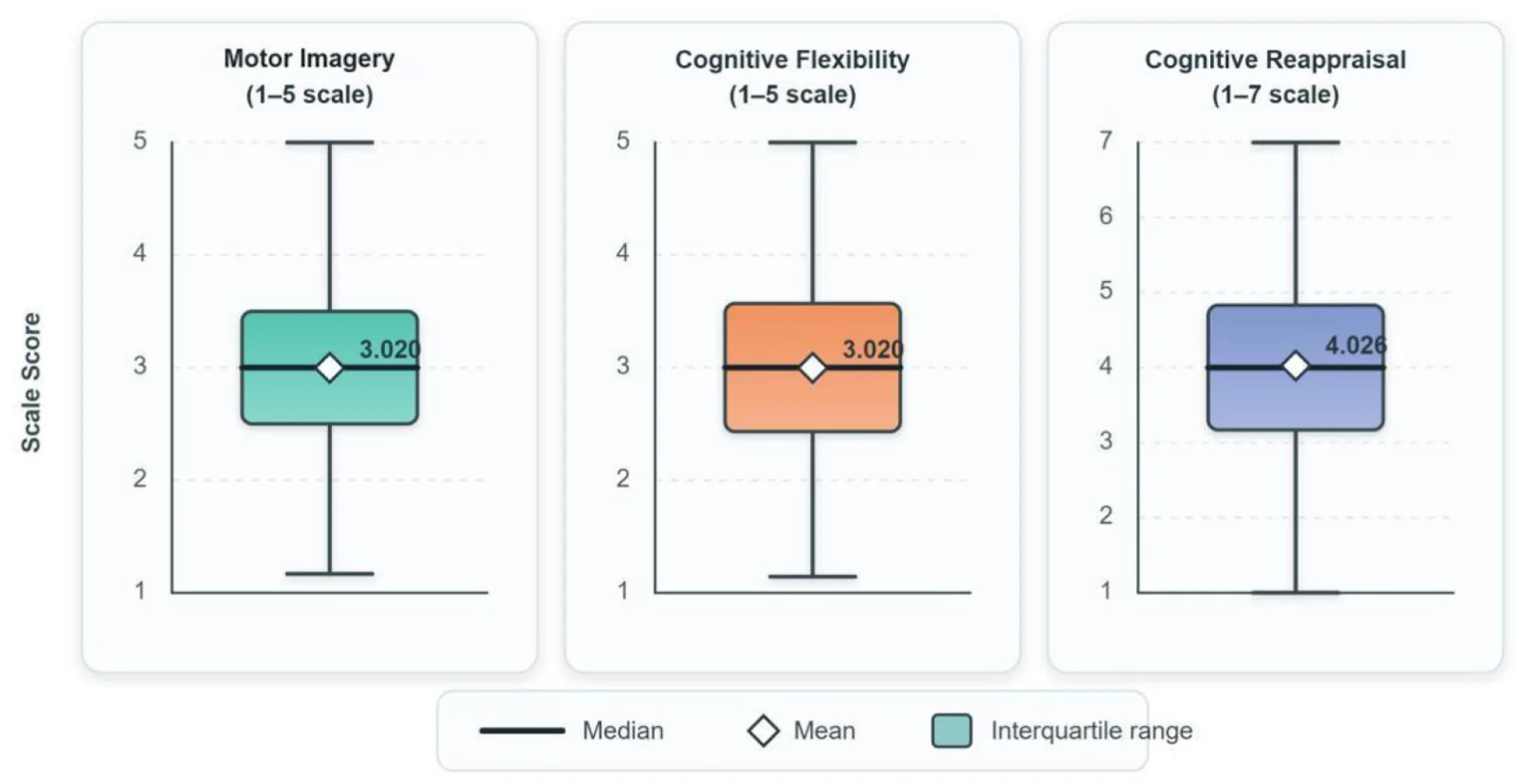
Boxplots of core variable distributions and outlier detection. The upper and lower boundaries of the box represent the third and first quartiles respectively, and the horizontal line inside the box represents the median. The upper and lower whiskers are determined according to the 1.5×IQR rule.

After data cleaning and verification, a final *N* = 1,070 valid and complete questionnaires were obtained, with no missing values in the dataset. The distribution of participants' basic demographic characteristics and dance training experience is shown in Table 1; the distribution of core variables and potential outlier detection are shown in Figure 1.

**Table 1:** Demographic and dance training characteristics distribution of the survey sample.

Variable	Category	Frequency (*n*)	Percentage (%)
Gender	Male	364	34.02
	Female	706	65.98
Highest education	High school and Below	59	5.51
	Associate's/vocational degree	152	14.21
	Bachelor's degree	755	70.56
	Master's degree and above	104	9.72
Dance training experience	Regular dance training experience	464	43.36
	No relevant dance training experience	606	56.64

*N* = 1070. The age range of participants was 18−35 years.

The boxplots are used to display the distributional characteristics of the core variables and assist in identifying potential anomalous observations, without automatically judging all observations beyond the whiskers as invalid data.

Harman's single-factor test was used to examine common method bias (CMB) for all 19 measurement items. Unrotated principal component analysis results showed that the variance explained by the largest extracted common factor was only 30.51%, far below the critical standard of 40%; the cumulative variance explained by the first 3 factors was 50.43 (see Table 2). These results suggest that common method bias is unlikely to pose a serious concern in the present study, although its presence cannot be entirely ruled out.

**Table 2:** Results of Harman's single-factor common method bias test.

Principal component no.	Initial eigenvalue	Variance explained (%)	Cumulative variance explained (%)
1	5.797	30.51	30.51
2	2.034	10.71	41.21
3	1.750	9.21	50.43

### 4.2 Internal consistency reliability of core measurements

Cronbach's α coefficient was used to test the internal consistency of each scale. The results showed: the α coefficient of the motor imagery subscale was 0.814, the cognitive flexibility scale was 0.773, and the cognitive reappraisal subscale was 0.852 (see Table 3 for details). The above results indicate that all measurement tools used in this study possess good internal consistency reliability, meeting the standards for further statistical inference.

**Table 3:** Internal consistency reliability of core measurements.

Core variable	Item range	No. of items	Cronbach's α	Judgment
Motor imagery	Q7—Q12	6	0.814	Good
Cognitive flexibility	Q13—Q19	7	0.773	Acceptable
Cognitive reappraisal	Q20—Q25	6	0.852	Good

### 4.3 Descriptive statistics and group comparisons of core variables

#### 4.3.1 Descriptive statistics of core variables

Table 4 shows the descriptive statistics and distribution characteristics of the three core variables. The mean scores for motor imagery and cognitive flexibility were both 3.020 (SD ranging between 0.758 and 0.823). The mean score for cognitive reappraisal was 4.026 (*SD* = 1.174). The absolute values of the skewness coefficients of the core variables' distributions were all less than 0.1, and the absolute values of the kurtosis coefficients were all less than 0.6, indicating that the data distribution presents a good normal approximation, meeting the basic assumptions of subsequent parametric tests.

**Table 4:** Descriptive statistics and distribution characteristics of core variables.

Core variable	M	SD	Minimum	Median	Maximum	Skewness	Kurtosis
Motor imagery	3.020	0.823	1.167	3.000	5.000	0.091	−0.54
Cognitive flexibility	3.020	0.758	1.143	3.000	5.000	−0.001	−0.42
Cognitive reappraisal	4.026	1.174	1.000	4.000	7.000	0.094	−0.21

*N* = 1070. Motor imagery and cognitive flexibility used a 1-−5 point scoring system, and cognitive reappraisal used a 1-−7 point scoring system.

#### 4.3.2 Comparison of core variables across different dance training experience groups

**Figure 2:**
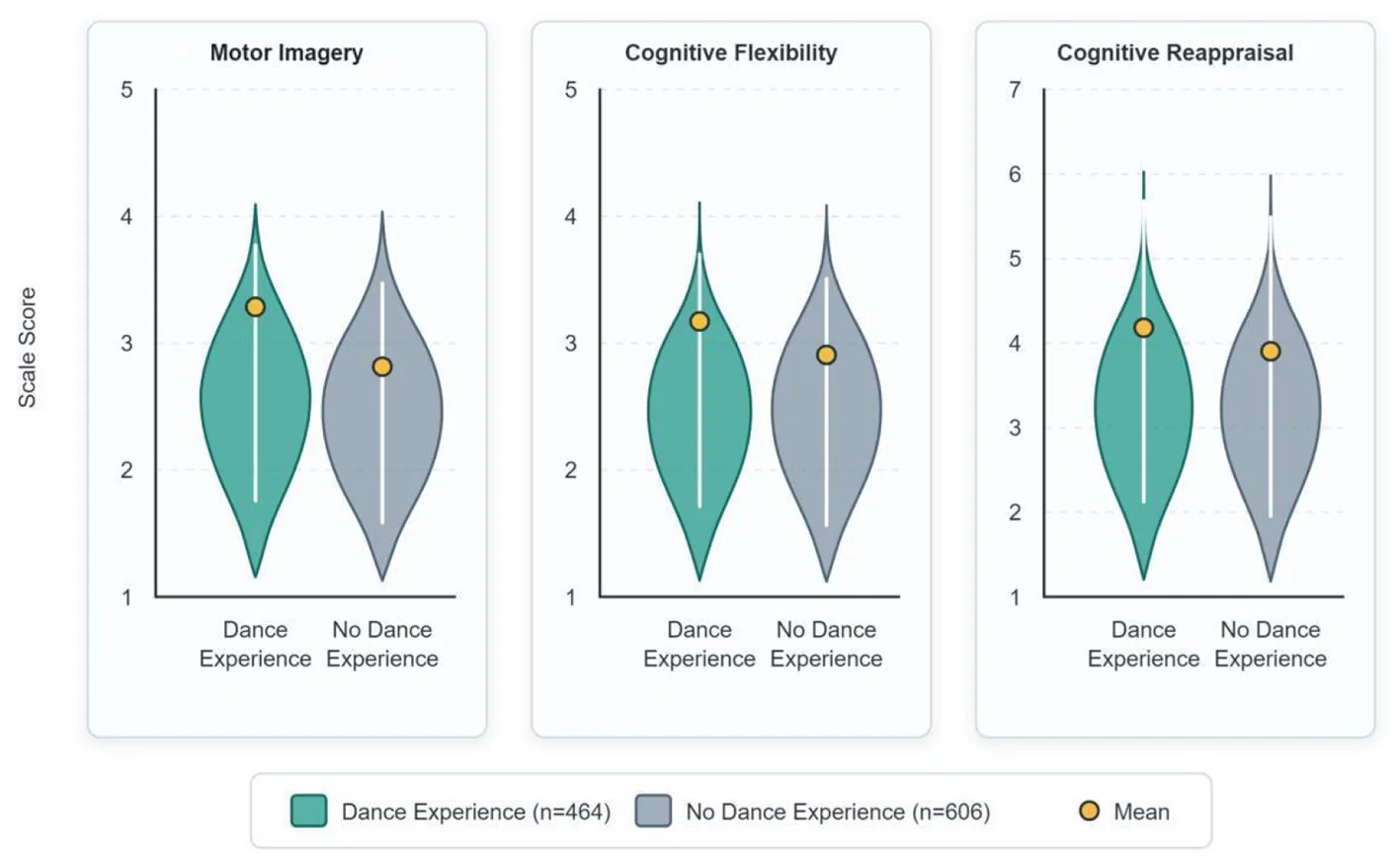
Violin plots of core variable distributions by different dance training experience groups. The violin plots display the distribution density of motor imagery, cognitive flexibility, and cognitive reappraisal scores for groups with and without dance training experience. The markers in the plots indicate the mean of each group.

In order to compare basic differences in core variables across different dance training experience groups, participants were divided into an “experienced group” and an “inexperienced group”. Since some variables had slight variance heterogeneity between groups (e.g., cognitive flexibility p=0.043), this study uniformly adopted Welch's independent samples t-test, which is more robust to heterogeneous variances. The distribution density comparison of variable scores between the two groups is shown in Figure 2.

The test results showed (see Table 5) that regarding motor imagery scores, the group with regular dance training experience (*M* = 3.286) was significantly higher than the inexperienced group (*M* = 2.817), *t*(1011.88) = 9.663, *p* < 0.001, with an effect size reaching an upper-middle level (Cohen's *d* = 0.594). In terms of cognitive flexibility and cognitive reappraisal scores, the experienced group was also significantly higher than the inexperienced group, both p < 0.001. These results verify hypothesis H1, indicating a significant statistical association between dance training experience and individuals' higher subjective action simulation capabilities and emotional cognitive regulation levels.

**Table 5:** Welch's independent samples test of core variables by different dance training experience groups.

Core variable	Experienced group (*n* = 464) *M* ±*SD*	Inexperienced group (*n* = 606) *M* ±*SD*	Mean Diff.	95% CI	Welch *t*	*df*	*p*	Cohen's *d*
Motor imagery	3.286 ± 0.775	2.817 ± 0.801	0.469	[0.374, 0.564]	9.663	1011.88	< 0.001	0.594
Cognitive flexibility	3.169 ± 0.717	2.907 ± 0.770	0.262	[0.172, 0.351]	5.735	1028.42	< 0.001	0.350
Cognitive reappraisal	4.180 ± 1.177	3.908 ± 1.158	0.272	[0.131, 0.414]	3.778	988.20	< 0.001	0.234

The mean difference is calculated as “experienced group minus inexperienced group”, with positive values indicating higher scores in the experienced group*; CI* is the 95% confidence interval of the mean difference.

#### 4.3.3 Within-group analysis of dance training

Gradient Association Characteristics of Training Duration and Frequency To further explore the fine-grained statistical association between dance training characteristics on variables related to embodied cognition, the study conducted a one-way analysis of variance (ANOVA) on the subpopulation reporting “regular dance training experience” (n = 464). The results are detailed in Table 6.

**Table 6:** Core variable scores and ANOVA results within different dance training duration and frequency groups (*n* = 464).

Variable category	Group	*N*	Motor imagery (M ±SD)	Cognitive flexibility (M ±SD)	Cognitive reappraisal (M ±SD)
Cumulative training duration	Under 1 year	98	3.08 ± 0.72	3.12 ± 0.73	4.15 ± 1.20
	1–3 years	136	3.22 ± 0.75	3.15 ± 0.69	4.12 ± 1.15
	3–5 years	115	3.35 ± 0.79	3.19 ± 0.70	4.21 ± 1.14
	More than 5 years	115	3.51 ± 0.78	3.22 ± 0.74	4.25 ± 1.21
	*F*_(3, 460)_		6.75^***^	0.48	0.39
	ηp2		0.042	0.003	0.002
	*Post-hoc* Test		>5 years > 1-3 years, < 1 year	—	—
Past 6-Month training frequency	Occasionally (< 1/week)	85	3.06 ± 0.74	3.11 ± 0.71	4.11 ± 1.16
	1–2 times/week	172	3.25 ± 0.78	3.17 ± 0.68	4.16 ± 1.18
	3–4 times/week	125	3.34 ± 0.75	3.18 ± 0.75	4.20 ± 1.22
	≥5 times/week	82	3.49 ± 0.81	3.21 ± 0.72	4.26 ± 1.13
	*F*_(3, 460)_		5.12^**^	0.41	0.35
	ηp2		0.032	0.002	0.002
	*Post-hoc* test		≥5 times > Occasionally	—	—

*n* represents the sample size of each subgroup. *Post-hoc* multiple comparisons used Bonferroni correction. ^**^ *p* < 0.01, ^***^ *p* < 0.001.

The analysis results showed that both cumulative training duration and training frequency in the past six months reported significant main effects on motor imagery scores. Combined with Bonferroni *post-hoc* comparisons, it can be seen that motor imagery ability presents a gradient upward trend with the increase of training intensity: in terms of duration, the “more than 5 years” group was significantly higher than the “under 3 years” groups; in terms of frequency, the “5 times and above per week” group was significantly higher than the occasional trainers. However, although cognitive flexibility and cognitive reappraisal scores showed a faint upward trend with increased training investment, the differences did not reach statistical significance.

The above results indicate a significant linear gradient association between dance experience and basic-level sensorimotor abilities (motor imagery), while higher-order cognitive and emotional reappraisal abilities did not vary significantly across different levels of training duration and frequency. These findings indicate a foundational inter-group advantage associated with dance experience, rather than a continuous dose-response relationship accumulating with practice volume.

### 4.4 Correlation and hierarchical regression analysis of core variables

#### 4.4.1 Correlation analysis of core variables

Pearson correlation analysis was used to examine the bivariate associations among core variables. The correlation coefficients (see Table 7) indicated that all three variables were significantly positively correlated with each other.

**Table 7:** Pearson correlation matrix of core variables.

Core variable	1	2	3
1. Motor imagery	1.000		
2. Cognitive flexibility	0.369^***^	1.000	
3. Cognitive reappraisal	0.392^***^	0.458^***^	1.000

*N* = 1070. Values in the table are Pearson correlation coefficients; ^***^ *p* < 0.001.

**Figure 3:**
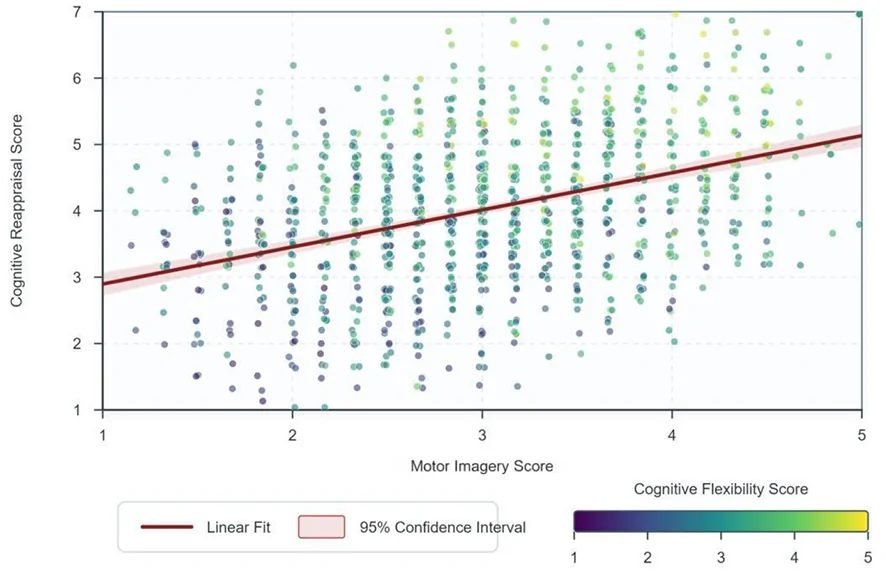
Scatterplot and linear fit of motor imagery and cognitive reappraisal. The horizontal axis represents the motor imagery score, and the vertical axis represents the cognitive reappraisal score; scatter colors indicate cognitive flexibility levels; the solid line represents the linear fitting trend, and the shaded area is the 95% confidence interval. Each scatter point corresponds to an observed score of a participant.

Specifically, motor imagery was significantly positively correlated with cognitive flexibility (*r* = 0.369, *p* < 0.001) and significantly positively correlated with cognitive reappraisal (*r* = 0.392, *p* < 0.001). There was also a moderate positive correlation between cognitive flexibility and cognitive reappraisal (*r* = 0.458, *p* < 0.001). A stable positive synergistic relationship exists among the three (scatter distribution and fitting trend seen in Figure 3), and no high collinearity risk was triggered, supporting hypothesis H2.

#### 4.4.2 Hierarchical multiple regression analysis

To test the independent statistical capabilities of motor imagery and cognitive flexibility on cognitive reappraisal, this study constructed a hierarchical multiple regression model. As shown in Table 8, Model 1 included demographic control variables such as gender, age, and education, and the overall model was not significant (*R*^2^ = 0.003, *F* = 1.150, *p* > 0.05). In Model 2, after further incorporating standardized motor imagery and cognitive flexibility variables, the model's explanatory power significantly improved (Δ*R*^2^ = 0.265, *p* < 0.001). The results show that after controlling for demographic variables, both motor imagery (β = 0.257, *p* < 0.001) and cognitive flexibility (β = 0.363, *p* < 0.001) independently and significantly positively predict an individual's cognitive reappraisal tendency.

**Table 8:** Results of hierarchical multiple regression analysis for cognitive reappraisal.

Predictor	Model 1 β	Model 1 *t*	Model 1 *p*	Model 2 β	Model 2 *t*	Model 2 *p*
Gender	0.016	0.515	0.607	0.025	0.951	0.342
Age	−0.033	−1.064	0.287	−0.003	−0.127	0.899
Education	−0.044	−1.437	0.151	−0.020	−0.752	0.452
Motor imagery	—	—	—	0.257	9.108	< 0.001
Cognitive flexibility	—	—	—	0.363	12.822	< 0.001
*R* ^2^	0.003			0.268		
Adjusted *R*^2^	0.000			0.265		
Δ*R*^2^	0.003			0.265		
Model *F*	1.150		0.328	78.014		< 0.001
Δ*F*	1.150		0.328	192.690		< 0.001

*N* = 1070, dependent variable is cognitive reappraisal. β in the table represents standardized regression coefficients. Model 1 included gender, age, and education; Model 2 further included motor imagery and cognitive flexibility. Gender was coded as *Male* = 0, *Female*=1; education was coded as an ordered level from 1 to 4.

### 4.5 Statistical mediation analysis of cognitive flexibility

Based on controlling for demographic variables, the PROCESS macro (Model 4) was further used to test the statistical mediation effect of cognitive flexibility between motor imagery and cognitive reappraisal, estimating the 95% confidence interval with 5,000 Bootstrap resamples.

**Figure 4:**
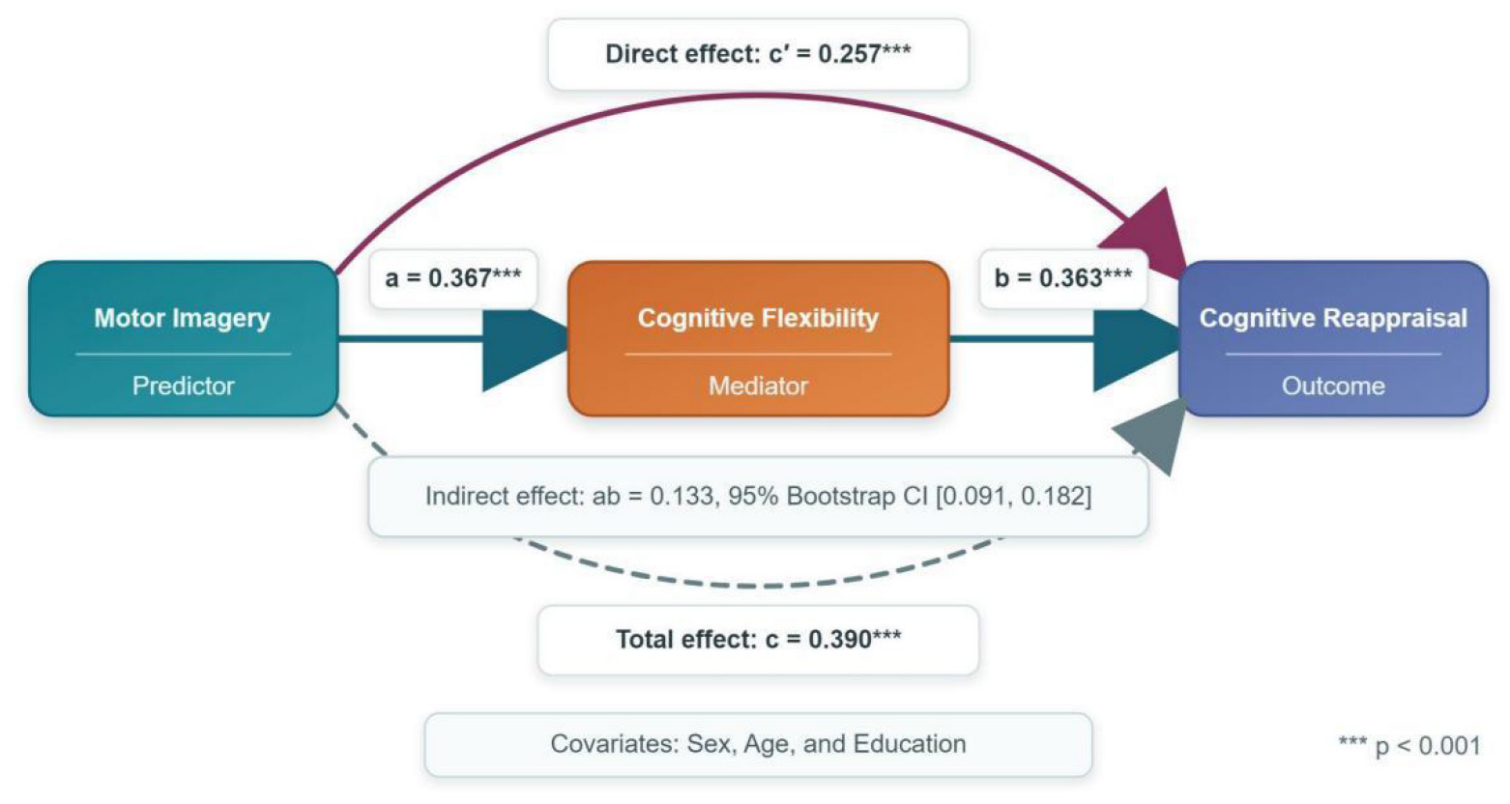
Statistical mediation model of cognitive flexibility between motor imagery and cognitive reappraisal. Path values represent standardized regression coefficients. *a* = effect of predictor on mediator; *b* = effect of mediator on outcome; *c* = total effect; *c*′ = direct effect. The model controlled for covariates including gender, age, and education. ^***^ *p* < 0.001.

The analysis results showed that motor imagery significantly positively predicts cognitive flexibility (*a* = 0.367, *p* < 0.001); and cognitive flexibility also significantly positively predicts cognitive reappraisal tendency (*b* = 0.363, *p* < 0.001). The total effect of motor imagery on cognitive reappraisal was significant (*c* = 0.390, *p* < 0.001). When cognitive flexibility was added as a mediator variable, the direct effect of motor imagery on cognitive reappraisal remained significant (*c*′ = 0.257, *p* < 0.001). The mediation effect test showed that the indirect effect of motor imagery on cognitive reappraisal through cognitive flexibility was significant (*ab* = 0.133), and the 95% Bootstrap CI was [0.091, 0.182] (not containing 0). The ratio of this indirect effect to the total effect was 34.11%. Research hypothesis H3 was verified, and the results supported the partial mediation role of cognitive flexibility in this path. The theoretical path model diagram is shown in Figure 4, and the statistical test details are in Table 9.

**Table 9:** Bootstrap test results for the statistical indirect effect of cognitive flexibility.

Effect type	Standardized effect	Relative proportion (%)	95% Bootstrap CI	Statistical judgment
Total effect *c*	0.390	100.00	—	*p* < 0.001
Direct effect *c'*	0.257	65.89	—	*p* < 0.001
Indirect effect *ab*	0.133	34.11	[0.091, 0.182]	CI does not contain 0

The number of Bootstrap resamples was 5,000. The model controlled for gender, age, and education. Motor imagery, cognitive flexibility, and cognitive reappraisal were *Z*-standardized before entering the model, thus the effects and their confidence intervals in the table are reported on a standardized scale.

## 5 Discussion

### 5.1 Main findings

Based on cross-sectional survey data from 1,070 young adults, this study systematically examined the structural relationship among dance training experience, motor imagery, cognitive flexibility, and emotion regulation strategies (cognitive reappraisal). The study results confirmed solid positive statistical associations among these variables.

#### 5.1.1 Inter-group differences and non-linear properties

Participants with regular dance training experience scored significantly higher in motor imagery, cognitive flexibility, and cognitive reappraisal than the inexperienced group. In particular, the fine-grained within-group analysis revealed a unique empirical feature: basic sensorimotor abilities (motor imagery) show a gradient effect of linear growth with the increase in training intensity, whereas higher-order cognitive and emotional reappraisal abilities exhibit a foundational inter-group advantage associated with regular training experience, rather than increasing continuously with training volume. This finding suggests a potential non-linear relationship regarding how complex movement training relates to different psychological functions. It implies that higher-order emotional and cognitive advantages may not stem solely from the continuous accumulation of physical movement intensity, but could be mutually facilitated by other comprehensive elements inherent in dance activities, such as musical rhythm feedback and social interaction.

#### 5.1.2 Positive structural associations among variables

Correlation and regression analyses confirmed the strong associative properties among motor imagery, cognitive flexibility, and cognitive reappraisal. Specifically, individuals with higher clarity in multisensory (visual and kinesthetic) motor imagery tend to demonstrate stronger cognitive flexibility in information processing (e.g., being better at breaking mental fixedness and generating alternatives when facing setbacks). At the same time, people with good foundations in these two areas are more inclined to invoke cognitive reappraisal strategies in their daily lives. This positive association among multiple variables reflects the intrinsic connection during the theoretical transfer from the sensorimotor representation level to higher-order emotion regulation strategies.

#### 5.1.3 Partial statistical mediation effect of cognitive flexibility

The results of the mediation model further revealed the statistical structural pathways among the variables. Data showed that motor imagery not only has a direct positive association with cognitive reappraisal but also exerts a significant indirect predictive effect on it through cognitive flexibility (the indirect effect accounting for approximately 34.11%). This result indeed supports a theoretical structural chain of “embodied action representation — cognitive flexible adaptation — emotional cognitive reconstruction”. Cognitive flexibility plays a key “bridge” role between lower-order embodied experiences and higher-order emotion regulation. The results of this study suggest: a robust capacity for clear dynamic spatial schemas and action representation effectively parallels individuals' flexible resource allocation in working memory; once this anti-rigidity “mental flexibility” is established, it provides sufficient prerequisite psychological resources for further constructing multi-level perspectives to reinterpret negative stressful events (i.e., implementing cognitive reappraisal). It should be emphasized that while this theoretical structural chain is compelling, it fundamentally reflects concurrent positive associations. Therefore, rather than concluding that dance training directly improves cognitive and emotional functions, it is more accurate to view these abilities as an intrinsically linked physical-psychological network.

### 5.2 Practical implications

The results of this study provide novel and solid empirical support for physical-mental synergistic health promotion programs in non-clinical settings. Faced with psychological stress and emotional distress common among young adults (such as college students and new employees), traditional psychological counseling relying mainly on verbal communication can sometimes trigger individual psychological defenses or be hindered by “psychological stigma”. This study suggests that comprehensive physical activities like dance can form positive connections with individuals' high-level emotion regulation networks (Chen et al., 2025; Prates et al., 2025; Zafeiroudi et al., 2025).

Therefore, in future mental health interventions and policymaking, universities or communities could develop specialized “embodied dance stress-reduction programs”. Such programs should break away from traditional sports models that merely pursue specific dance beat speeds or difficult cardiopulmonary indicators, and instead emphasize “internal action simulation (imagery arousal), spatial self-positioning perception, and flexible shifting of multisensory perspectives” (Yu et al., 2025). By implicitly integrating prerequisite training of cognitive functions into fun, highly compliant daily physical activities without any “treatment stigma”, an effective emotion regulation resource pool can be established for young groups (Mahindru et al., 2023; Millman et al., 2021; Singh et al., 2023).

### 5.3 Research limitations and future prospects

Although this study has certain theoretical and practical significance, there are still the following limitations:

First, this study adopted a cross-sectional design and cannot infer causal relationships or chronological order among variables. Particularly, due to self-selection bias, this study cannot rule out the possibility of reverse causality: that is, whether individuals possessing higher internal visual imagery capabilities and cognitive flexibility are naturally more inclined toward and more easily maintain long-term dance training. Future studies need to adopt longitudinal tracking designs or randomized controlled trials (RCT) to further confirm the exact causal mechanisms of somatic training and higher-order cognitive changes.

Second, this study used convenience sampling and snowball sampling through an online survey, which may lead to certain limitations in sample representativeness and introduce selection bias, thereby limiting the generalizability of the current findings.

Third, the present research design lacked sufficient objective control over several confounding variables. Factors such as participants' overall level of daily physical activity, baseline mental health status, previous athletic background, specific types of dance engaged in, and exact intensity of physical training were not rigorously accounted for. These unmeasured variables may inflate or confound the observed relationships. Future research needs to test the external validity of the conclusions in a broader, more evenly distributed demographic sample while rigorously controlling for these covariates.

Fourth, all main variables were assessed using self-report questionnaires and may therefore be susceptible to social desirability effects and common method bias. Although the results of Harman's single-factor test suggest that common method bias is unlikely to pose a serious concern in the present study, its potential influence cannot be entirely ruled out. Future research could adopt multi-source and multi-method assessments by integrating self-report data with objective behavioral measures and neuroimaging or neurophysiological techniques, such as functional magnetic resonance imaging (fMRI) and electroencephalography (EEG) (Yang et al., 2023). Such a multi-method approach would reduce reliance on self-reported data and provide more objective evidence of the behavioral and neural mechanisms involved, particularly the role of frontoparietal executive-control networks associated with the “neural reuse principle.”

Finally, this study only roughly controlled for the duration and frequency of dance training, without detailing the potential heterogeneity of specific dance genres and movement intensities. For instance, highly stylized dance genres (such as classical ballet) and free dance genres highly dependent on improvisation and environmental interaction (such as modern dance, hip-hop) may have fundamentally different requirements and embodied shaping paths for individuals' cognitive flexibility, providing broad research space for subsequent, more precise variable control and comparative experiments.

## 6 Conclusion

Based on a large-sample cross-sectional survey, this study systematically explored the structural associations among dance training experience, motor imagery, cognitive flexibility, and cognitive reappraisal strategies in young adults. The results showed that individuals with regular dance training experience demonstrated significantly higher levels in the clarity of internal action simulation, cognitive adaptive flexibility, and active utilization of cognitive reappraisal strategies. Further data models revealed that higher motor imagery ability demonstrates an independent and direct positive correlation with an individual's emotional cognitive reappraisal tendency, as well as a significant statistical indirect link through cognitive flexibility. This path aligns with a theoretical hierarchical framework conceptually linking “embodied action representation”, passing through “cognitive flexible adaptation”, and ultimately serving “emotional cognitive reconstruction”. This indicates that psychological processing capabilities derived from the body and sensorimotor simulation levels are closely associated with the brain's cognitive shifting flexibility when dealing with external changes, thereby indirectly correlating with individuals in constructing more positive emotion regulation frameworks. This study provides robust large-sample quantitative empirical evidence for embodied cognition theory—that lower-order sensorimotor experiences can form progressive associations with higher-order emotional and cognitive control networks. By viewing these physical and psychological capacities as an interconnected network, these findings provide theoretical support for comprehensive physical activity habits and mental health regulation, pointing out a highly potential innovative direction for the education and public health fields to develop new daily intervention programs that combine physical activity with emotional buffering functions for youth emotional wellbeing.

## Data Availability

The original contributions presented in the study are included in the article/Supplementary material, further inquiries can be directed to the corresponding author.
