# Supplementary material for: A study on the structural relationship among dance training experience, motor imagery, cognitive flexibility, and emotion regulation strategies in young adults
Source: Front Psychol. 2026 Sep 4;17:1956871. doi: 10.3389/fpsyg.2026.1956871 (PMC13585626; doi:10.3389/fpsyg.2026.1956871)
Supplement: Supplementary file 1 [file Data_Sheet_1.ZIP › Supplementary Material 2 - Data Codebook.docx]

Data Codebook (Data Codebook)

Research Project Name: A Study on the Structural Relationship among Dance Training Experience, Motor Imagery, Cognitive Flexibility, and Emotion Regulation Strategies in Young Adults
Sample Size: *N* = 1070 (Cleaned valid data)

**I. Questionnaire Screening Questions and Basic Demographic Variables (Demographics)**

| Variable Name (Variable) | Label/Meaning (Label) | Assignment Rules/Option Definitions (Values/Coding) | Missing Value Description |
| --- | --- | --- | --- |
| Q0 | Sample Validity (Screening) | 1 = Yes, 18-35 years old valid sample (Note: Invalid samples with a value of 2 have been completely removed during the cleaning phase) | No missing values |
| Q1_Age | Actual Age (Age) | Continuous variable (18 ~ 35), Unit: years | No missing values |
| Q2_Sex | Gender (Gender) | 1 = Male (Male) 2 = Female (Female) | No missing values |
| Q3_Edu | Highest Education (Education) | 1 = High school and below 2 = Associate’s/Vocational degree 3 = Bachelor’s degree 4 = Master’s degree and above | No missing values |

**II. Dance Training Experience (Dance Experience)**

| Variable Name (Variable) | Label/Meaning (Label) | Assignment Rules/Option Definitions (Values/Coding) | Missing Value Description |
| --- | --- | --- | --- |
| Q4_Dance | Whether has dance experience (Dance Exp. Group) | 1 = Yes (Has training experience) 2 = No (No training experience) | No missing values |
| Q5_DanceTime | Cumulative Training Duration (Dance Duration) | 1 = Less than 1 year 2 = 1~3 years 3 = 3~5 years 4 = More than 5 years | System Missing: When Q4=2, this column is blank. |
| Q6_DanceFreq | Weekly Training Frequency in the past six months (Dance Frequency) | 1 = Occasionally, less than once a week 2 = 1~2 times a week 3 = 3~4 times a week 4 = 5 times or more a week | System Missing: When Q4=2, this column is blank. |

**III. Motor Imagery Ability (VMIQ-2 Short Version)**

Scoring rules: 1=No image at all, 5=As clear as seeing it in person. All are forward-scored items, higher scores indicate clearer imagery.

| Variable Name (Variable) | Label / Sub-scale (Label / Sub-scale) | Assignment Rules/Option Definitions (Values/Coding) |
| --- | --- | --- |
| Q7 | Continuous forward jumping (External visual imagery) | 1=Not at all … 5=Extremely clear |
| Q8 | Clapping to music (External visual imagery) | 1=Not at all … 5=Extremely clear |
| Q9 | Single-leg balance (External visual imagery) | 1=Not at all … 5=Extremely clear |
| Q10 | Leg contraction and force sensation (Kinesthetic imagery) | 1=Not at all … 5=Extremely clear |
| Q11 | Regaining center of gravity when about to fall (Kinesthetic imagery) | 1=Not at all … 5=Extremely clear |
| Q12 | Sense of bodily coherence in skilled movements (Kinesthetic imagery) | 1=Not at all … 5=Extremely clear |
| VMIQ_Mean (To be created) | Total mean score of motor imagery | Formula: = MEAN(Q7…Q12) |

**IV. Cognitive Flexibility (CFI Short Version)**

Scoring rules: 1=Strongly disagree, 5=Strongly agree. Note that it includes Reverse-scored items.

| Variable Name (Variable) | Label / Sub-scale (Label / Sub-scale) | Scoring Direction |
| --- | --- | --- |
| Q13 | Considering multiple options before making decisions (Alternatives) | Forward (1=1 … 5=5) |
| Q14 | Coming up with several methods when in difficulty (Alternatives) | Forward (1=1 … 5=5) |
| Q15 | Quickly finding new ways when unplanned (Alternatives) | Forward (1=1 … 5=5) |
| Q16 | Willing to listen to entirely new perspectives (Alternatives) | Forward (1=1 … 5=5) |
| Q17 | Hard to change fixed ways under stress (Control and Rigidity) | Reverse scoring (1=5, 2=4, 3=3, 4=2, 5=1) |
| Q18 | Frustrated and hard to think of alternatives when rejected (Control and Rigidity) | Reverse scoring (1=5, 2=4, 3=3, 4=2, 5=1) |
| Q19 | Getting stuck in a dead end when frustrated (Control and Rigidity) | Reverse scoring (1=5, 2=4, 3=3, 4=2, 5=1) |
| CFI_Mean (To be created after processing) | Total mean score of cognitive flexibility | Formula: = MEAN(Q13, Q14, … Q19 reverse) |

**V. Emotion Regulation Strategies (ERQ Cognitive Reappraisal Dimension)**

Scoring rules: 1=Strongly disagree, 7=Strongly agree. All are forward-scored items. Higher scores indicate more frequent use of reappraisal strategies.

| Variable Name (Variable) | Label/Meaning (Label) | Assignment Rules/Option Definitions (Values/Coding) |
| --- | --- | --- |
| Q20 | Changing thoughts for positive emotions | 1=Strongly disagree … 7=Strongly agree |
| Q21 | Changing perspectives to reduce negative emotions | 1=Strongly disagree … 7=Strongly agree |
| Q22 | Calmly rethinking under stress | 1=Strongly disagree … 7=Strongly agree |
| Q23 | Changing views on situations to control emotions | 1=Strongly disagree … 7=Strongly agree |
| Q24 | Shifting focus to reduce negative experiences | 1=Strongly disagree … 7=Strongly agree |
| Q25 | Finding positive aspects to make oneself happy | 1=Strongly disagree … 7=Strongly agree |
| ERQ_Mean (To be created) | Total mean score of cognitive reappraisal strategies | Formula: = MEAN(Q20…Q25) |
