# Supplementary material for: A study on the structural relationship among dance training experience, motor imagery, cognitive flexibility, and emotion regulation strategies in young adults
Source: Front Psychol. 2026 Sep 4;17:1956871. doi: 10.3389/fpsyg.2026.1956871 (PMC13585626; doi:10.3389/fpsyg.2026.1956871)
Supplement: Supplementary file 1 [file Data_Sheet_1.ZIP › Supplementary Material 1 - Questionnaire.docx]

Questionnaire on Movement Experience and Thinking Styles of Young Adults

Dear participant, hello!

This is a purely academic study on physical movement experience, motor imagery ability, and daily thinking styles. Your valuable feedback is crucial to our research. This questionnaire is completely anonymous and takes about 3-5 minutes to complete. All data will be used solely for overall statistical analysis, and personal privacy will never be compromised. This study follows the principle of voluntariness, and you can withdraw at any time. This questionnaire is only for young adults aged 18 to 35. By clicking “Agree” and continuing, you indicate that you have read and agreed to participate in this study. Thank you for your support!

**Q0. Is your current actual age between 18 and 35 years old? (Single choice, screening question)**

Yes, I am between 18 and 35 years old (Click to proceed to the main questionnaire)

No, I am under 18 or over 35 years old (Click to end the questionnaire immediately)

**Part 1: Basic Information and Movement Experience**

**Q1. Your actual age is:** ___ years old (Fill in the blank)

**Q2. Your gender: (Single choice)**

Male

Female

**Q3. Your highest level of education: (Single choice)**

High school and below

Associate’s / Vocational degree

Bachelor’s degree

Master’s degree and above

**Q4. Do you have any systematic or regular dance training experience? (Including professional study, long-term amateur training classes, university dance clubs, etc.) (Single choice, skip logic)**

Yes (Please continue to answer Q5, Q6)

No, I have barely had any contact / have not learned in such organizations (Skip directly to Q7)

**Q5. [If “Yes” to the previous question] Approximately how long is your cumulative time of receiving systematic dance training? (Single choice)**

Less than 1 year

1-3 years

3-5 years

More than 5 years

**Q6. [If “Yes” to the previous question] In the past six months, what is your average weekly frequency of participating in dance training/practice? (Single choice)**

Occasionally, less than once a week

1-2 times a week

3-4 times a week

5 times or more a week

**Part 2: Motor Imagery Ability**

[Instructions] The following questions will describe some specific movements. Please close your eyes, try to imagine the image or feeling in your mind, and rate it based on its vividness in your mind. (1=No image/sensation at all; 2=Vague and dim; 3=Moderately clear; 4=Relatively clear; 5=As clear as seeing/feeling it in person)

Please rate (1-5):
Q7. (Watching yourself like watching a video) Imagine yourself jumping forward continuously. [ 1 ] [ 2 ] [ 3 ] [ 4 ] [ 5 ]
Q8. (Watching yourself like watching a video) Imagine yourself clapping your hands to the rhythm immediately after hearing a complex rhythm. [ 1 ] [ 2 ] [ 3 ] [ 4 ] [ 5 ]
Q9. (Watching yourself like watching a video) Imagine yourself standing on one leg, trying hard to maintain body balance. [ 1 ] [ 2 ] [ 3 ] [ 4 ] [ 5 ]
Q10. (Not watching the image, but feeling the muscles) Imagine the feeling of your calf and foot muscles contracting and exerting force at the moment of jumping upward. [ 1 ] [ 2 ] [ 3 ] [ 4 ] [ 5 ]
Q11. (Not watching the image, but feeling the muscles) Imagine the muscle feeling of your body suddenly tensing up trying to regain the center of gravity when you are about to fall. [ 1 ] [ 2 ] [ 3 ] [ 4 ] [ 5 ]
Q12. (Not watching the image, but feeling the muscles) Imagine the sense of bodily coherence when smoothly completing a highly skilled set of movements. [ 1 ] [ 2 ] [ 3 ] [ 4 ] [ 5 ]

**Part 3: Daily Thinking Style**

[Instructions] Please read the following descriptions and choose based on how well they match your real situations in daily life. Please answer based on your first instinct. (1=Strongly disagree, 2=Somewhat disagree, 3=Unsure/Neutral, 4=Somewhat agree, 5=Strongly agree)

Please rate (1-5):
Q13. I usually consider multiple possible options before making a decision. [ 1 ] [ 2 ] [ 3 ] [ 4 ] [ 5 ]
Q14. When encountering difficulties, I can come up with several different ways to solve them. [ 1 ] [ 2 ] [ 3 ] [ 4 ] [ 5 ]
Q15. When something does not go as planned, I can quickly adjust and find a new way. [ 1 ] [ 2 ] [ 3 ] [ 4 ] [ 5 ]
Q16. I am willing to listen to entirely new perspectives on viewing things from people who are different from me. [ 1 ] [ 2 ] [ 3 ] [ 4 ] [ 5 ]
Q17. When I am in a stressful situation, I find it difficult to change my fixed way of looking at it. [ 1 ] [ 2 ] [ 3 ] [ 4 ] [ 5 ]
Q18. When others point out that my idea won’t work, I feel frustrated and find it hard to think of alternatives. [ 1 ] [ 2 ] [ 3 ] [ 4 ] [ 5 ]
Q19. When encountering setbacks, I often get stuck in the dead-end of “there is only one way, but I can’t do it”. [ 1 ] [ 2 ] [ 3 ] [ 4 ] [ 5 ]

**Part 4: Emotion Regulation Habits**

[Instructions] The following questions aim to understand your habitual ways of managing your inner feelings in daily life. Please select your level of agreement based on your actual situation. (1=Strongly disagree, 2=Somewhat disagree, 3=Slightly disagree, 4=Neutral/Unsure, 5=Slightly agree, 6=Somewhat agree, 7=Strongly agree)

Please rate (1-7):
Q20. When I want to feel more positive emotion (such as joy or amusement), I change what I’m thinking about. [ 1…7 ]
Q21. When I want to feel less negative emotion (such as sadness or anger), I change the way I’m thinking about the situation. [ 1…7 ]
Q22. When I’m faced with a stressful situation, I make myself think about it in a way that helps me stay calm. [ 1…7 ]
Q23. I control my emotions by changing the way I think about the situation I’m in. [ 1…7 ]
Q24. When I want to reduce the negative emotional experience I am having, I try to shift the focus in my mind. [ 1…7 ]
Q25. Even in a bad situation, as long as I want to feel happier, I can think about the positive aspects. [ 1…7 ]

You have completed all the questions! Thank you again for your valuable time and sincere answers, which are of great help to this research. Wishing you a happy life!
